# Effect of cerium lanthanide on Hela and MCF-7 cancer cell growth in the presence of transferring

**Published:** 2010

**Authors:** A.A. Palizban, H. Sadeghi-aliabadi, F. Abdollahpour

**Affiliations:** 1 *Department of Clinical Biochemistry and Isfahan Pharmaceutical Sciences Research Center, School of Pharmacy and Pharmaceutical Sciences, Isfahan University of Medical Sciences, Isfahan, I.R.Iran*; 2 Department of Medicinal Chemistry, School of Pharmacy and Pharmaceutical Sciences, Isfahan University of Medical Sciences, Isfahan, I.R.Iran

**Keywords:** Cytotoxicity, Cerium, Transferrin, Hela cell line, MCF-7 cell line, MTT assay

## Abstract

The anti-cancer activity of metal ions in the lanthanide group is being considered recently. It has been reported that cerium salts might stimulate the metabolism and therefore, produce anti-cancer effects. However, little is known about the effects of protein-cerium complex in controlling cancer cell growth. The aim of the present study was to elucidate the possible pathways for the cytotoxic effect of cerium in the presence of apo-transferrin on two cancer cell lines (Hela and MCF-7), that express transferrin receptors 3-4 fold higher than normal cells. The effect of different concentrations of cerium (0.1, 1, 10, 100 μM) in the presence and absence of transferrin for 48 h and 72 h incubation periods (37°C, 5% CO2 and 95% humidity) was studied using the MTT assay. The results showed that cerium has a cell-proliferation inhibitory activity which is significantly increased by transferrin protein. Compared with the direct treatment of cancer cells with cerium, the presence of transferrin assisted inhibition of cell proliferation by 20% and 40% in Hela and MCF-7 cells, respectively. Though apo-transferrin could lightly induce cell growth particularly in MCF-7 cells by itself, this phenomenon could not overcome the cerium-protein cell-proliferation inhibition activity. In conclusion, our results indicate that at a certain concentration, the cerium compounds could be possibly involved in the control of cell proliferation and inhibiting the growth of cancer cells.

## INTRODUCTION

The abnormal and unregulated cell growth, a factor that causes cancer cells, is characterized by damaging DNA or mutations to genes that encode proteins controlling cell division(1). These mutations may be caused by radiation, chemicals or physical agents, which are called carcinogens, or by certain viruses that can insert their DNA into the human genome. It is more than 50 years that scientists have spent their time on gathering data across hospital, local area, and even country boundaries, as a way to study the interdependence of environmental and cultural factors on cancer incidence(2,3). Cancer can be treated by many methods such as surgery, chemotherapy and radiation therapy. The choice of therapy depends upon the location and grade of the tumor and the stage of the disease, as well as the general state of the patient. Most of the chemotherapeutic agents affect DNA.

Trace elements and ultra trace elements have functional roles in most organisms and cells. In humans, the role of most elements such as iron is known and the physiological function of them has been well defined. There is fast moving research on lanthanides and their interrelations with bio-systems to understand their functional roles in biology and medicine(4,5). Lanthanides have been suggested for the treatment of a series of diseases and for diagnosis by magnetic resonance imaging. Recent studies have shown that lanthanides could act as scavengers of free radicals and therefore, protect cells and tissues from oxidative stress-induced injury. Evidently, the intracellular accumulation is very important in these cases(6). The ability of lanthanides such as cerium and their compounds to inhibit cancer cell proliferation would be of great interest in cancer chemotherapy. Cerium could be a good candidate to be studied if it has cytotoxic effect on cancer cells. Based on one reports, the cerium iodide solution called “introcid”, when injected into cancer patients during the early days of cancer chemotherapy, could shrink tumors more effectively than other iodine compounds. It was suggested that it was the cerium and not the iodine that inhibited the growth of cancer cells(7). Therefore, cerium complexes are apparently being developed as cancer drugs.

**Scheme 1:**
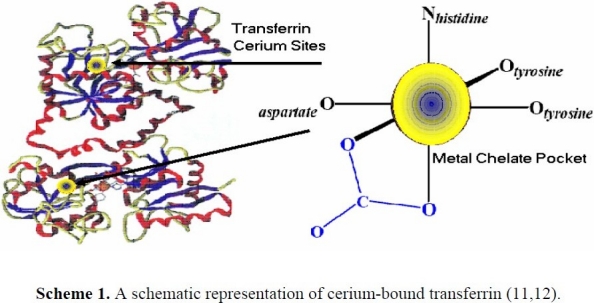
A schematic representation of cerium-bound transferrin (11,12).

The coordination chemistry of cerium could be somewhat similar to those of lanthanum (La) because they belong to the same group, but the coordination chemistry of cerium is very similar to iron(7,8). Therefore, due to biochemical similarities of cerium ion and ferric ion, particularly regarding binding protein and chelating binding sites, the iron physiological roles bear some resemblance to cerium(9,10). Transferrin is a plasma protein necessary for iron metabolism. It could also be an important protein for the metabolism of other metals like lanthanides. Therefore, cancer cells could uptake lanthanides via the transferrin receptor(11–13). Scheme 1 shows the proper binding sit of cerium on apo-transferrin protein.

Recent studies have shown that cancer cells are dependent on iron for their survival. Cerium ions could occupy the apo-transferrin binding sites distinct from iron which enables this element to consequently substitute iron and generate a cerium-transferrin complex. This situation could facilitate cells to uptake cerium instead of iron which is toxic to cells and would disturb the cell-proliferation network resulting in retardation of cell growth(14). Earlier studies on cell level and animal tests indicated that a high dose of lanthanide ion kills the cells by the cytotoxic behavior or the damaging effect. Necrosis or apoptosis are two different ways that anti-cancer agents kill tumor cells or inhibit tumor growth. A study on rat skin fibroblasts demonstrated that CeCl_3_ (10^-5^M) could induce apoptosis with a positive dose-dependence. The molecular mechanism of apoptosis is not very clear now. The possible pathways leading to lanthanide-induced apoptosis might be the increased intracellular ion concentration, followed by activation of endonuclease and protein kinase, DNA cleavage and apoptosis-related gene expressions(15). The aim of the present study was to underestand possible mechanisms for cytotoxic activity of cerium in the presence of apo-transferrin in Hela and MCF-7 cell lines which expresses transferrin receptors more than normal cells(16–18)

## MATERIALS AND METHODS

### 

#### Materials

RPMI 1640 medium was purchased from Gibco, Scotland. Tissue culture plastics were purchased from Nunc, Roskilde, Denmark. The cerium sulfate, was purchased from Merck, Germany. MTT (3-(4,5-dimethylthia-zol-2-yl)-2,5-diphenyl tetrazolium bromide) and DMSO were purchased from Sigma, USA. Phosphate-buffered saline (PBS) was purchased from Merck, Germany. The MCF-7 and Hela cell lines were purchased from Pasteur Institute, Tehran, Iran.

#### Cell culture

The Hela (a human epithelial cervical cancer) cell line and MCF-7 (Human breast adenocarcinoma) cell line were cultured at 37°C with a humidified incubator, 5% CO2, in RPMI 1640 medium containing 10% fetal calf serum and antibiotics (50 U/ml penicillin and 50 μg/ml streptomycin)(19).

#### MTT cell viability assay

MTT assay is based on a reaction between mitochondrial dehydrogenase enzymes from viable cells with the tetrazolium rings of MTT, a yellow reagent, to produce dark blue formazan crystals. These crystals are impermeable to cell membranes, thus resulting in its accumulation within healthy cells. Solubilisation of the cells by adding a detergent results in the liberation of the crystals, which are solubilized. The number of the surviving cells is correlated to the level of the formazan product which is generated(20,21). The color can then be quantified using the colorimetric assay. The results can be read on a multi-well scanning spectrophotometer (ELISA reader).

#### In vitro assays

To determine the effect of cerium on cell viability in the presence and absence of apo-transferrin, a rapid colorimetric MTT assay was used. The *in vitro* assays were divided into three main sections; each of them was used to determine: a) The correlation between the number of viable cells and resultant absorbance at 540 nm i.e. plotting standard curves. b) *In vitro* growth characteristics of cell lines by plotting curves to correlate absorbencies of viable cells against times (0, 24, 48, 72 and 96 h), i.e. plotting growth curves. c) Finally, chemosensitivity characteristics of cell lines against serial cerium concentrations in the presence and absence of apo-transferrin, via plotting relevant curves.

#### In vitro cytotoxicity assay

a) Assays were carried out in 96-well culture plates. The cells (10^5^) were allowed to settle by incubating the plates for 24 h before addition of the cerium and apo-transferrin solutions. After 48 h and 72 h continuous exposure to the compounds at 37 °C, the plates were analyzed for cell viability using MTT assay. The effect of cerium concentrations with and without apo-transferrin (1 nM) was assayed in 8 wells and within three independent experiments. The percentage of cell survival against cerium concentration was calculated by the following equation for Hela and MCF-7 cell line.

$$\%\mathrm{Cell}\;\mathrm{Survival}\;=\;\frac{\left(A_{t}-A_{b}\right)}{\left(A_{c}-A_{b}\right)}\;\times \;100$$

A_t_: Mean absorbance of the test compound

A_b_: Mean absorbance of the blank

A_c_: Mean absorbance of the negative control

b) Hela and MCF-7 cells were detached with trypsin (0.25%). The treated cells were counted and resuspended to a final concentration of 1×10^5^ cells per ml. From both cell lines, 180 μl of cell suspensions was added to each well of a 96-well plate. After 24 h of incubation, when cells were in the early exponential growth, both cell lines were treated as follows: a) As a blank, the Hela and MCF-7 cells received 200 μl complete medium (n=3), b) For sulfate control, the cells received 180 μl complete medium plus 20 μl sodium sulfate (n=3), c) To make apo-transferrin protein control (n=3), the cells received 180 μl complete medium plus 20 μl apo-transferrin (1 nM), d) and finally, the Hela and MCF-7 cell lines were treated with 180 μl and 160 μl complete medium plus a serial concentration of cerium (20 μl of 0.1, 1, 10 and 100 μM cerium sulfate solution) in the absence of apo-transerrin (n=3) and the presence of apo-transferrin (20 μl of 1 nM, n=3). The plates were incubated for 48 and 72 h. After that 20 μl MTT (5 mg/ml in PBS) was added to each well and incubated for another 3 h. Then the media was carefully removed and 150 μl of DMSO was added to each well to dissolve the blue formazan product. The absorbance of this product was measured at 540 nm, using ELISA plate reader (Stat Fax™® 2100 Microplate Reader, USA).

#### Statistical analysis

The statistical significance test applied to the data sets in this study was Student’s t-test for the comparison of two means.

## RESULTS

**Fig. 1:**
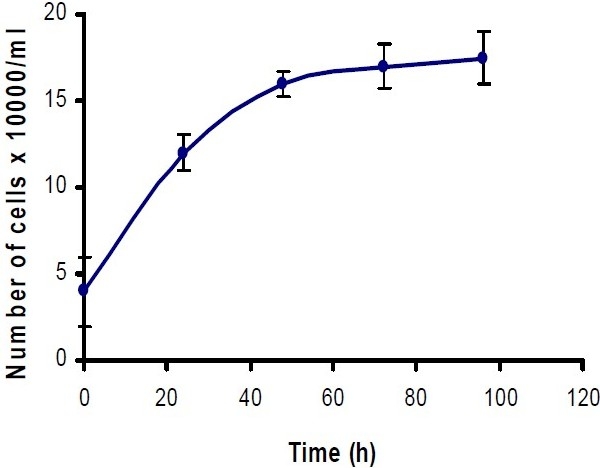
Hela cell growth curve assay during 96 h incubation period. Each point represents the number of cells against time. The results are the repeats of eight experiments in each day; cell viability was assessed using MTT assay (n=8). Data are presented as the mean ± SD.

**Fig. 2:**
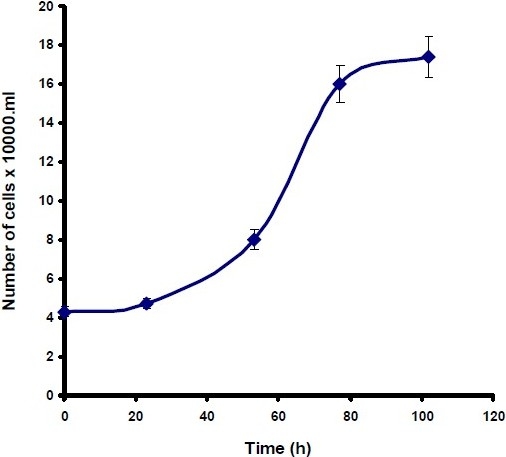
MCF-7 cell growth curve assay during 96 h incubation period. Each point represents the number of cells against time. The results are the repeats of eight experiments in each day; cell viability was assessed using MTT assay (n=8). Data are presented as the mean ± SD.

To investigate the effect of cerium on the proliferation of Hela and MCF-7 cells, in the presence and absence of apo-transferrin, the growth prospect of cancer cells was studied. To practically find the linearity of growth rate over the cell concentration range, a correlation study was performed for both cell lines by the method of linear regression analysis. The linearity of cell numbers against the absorbance of formazan product was observed at 540 nm by the MTT assay (n=3) for both cell lines. Regarding *in vitro* chemosensitivity assessment of cytotoxic agents against cancer cells, it is initially important to determine whether the cells are actively proliferating during the period of chemical administration, and to identify the growth characteristics of each cell line individually. It is also important to know if anti-cancer activity of cerium is dependent on the cancer cell type. Therefore, in this study the cells were initially counted, and then cultured using 96-well plates. The cells were incubated at 37° and at daily intervals. The number of viable cells were determined by the MTT assay (n=8). Fig. 1 and 2 show representative growth curves of Hela and MCF-7 cells, respectively in their logarithmic phase after 24 h.

**Fig. 3:**
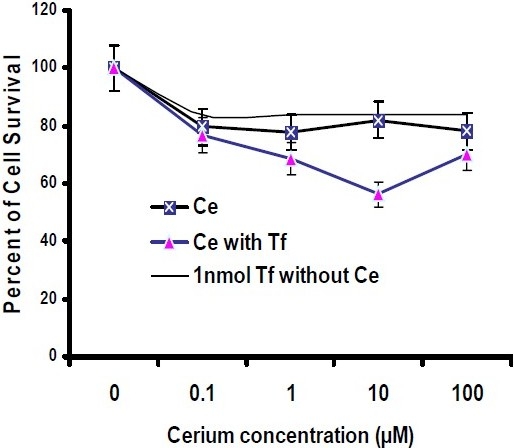
Percentage of Hela cell survival after treatment with different concentrations of cerium (0, 0.1, 1, 10 and 100 μM), incubated for 48 h in the presence or absence of transferrin (1 nM). The experiment in the absence and presence of transferrin is marked as 
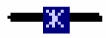
 and 
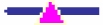
, respectively (n=8).

**Fig. 4:**
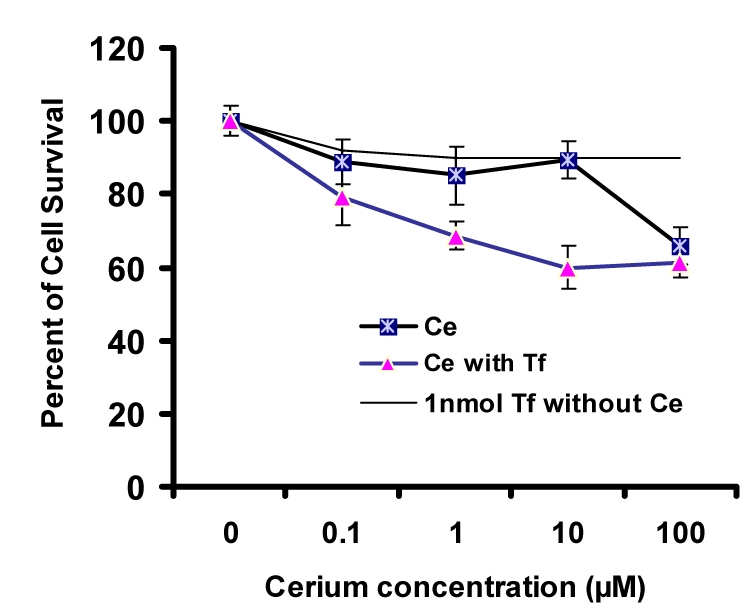
Percentage of Hela cell survival after treatment with different concentrations of cerium (0, 0.1, 1, 10 and 100μM), incubated for 72 h in the presence or absence of apo-transferrin (1 nM). The experiment in the absence and presence of transferrin is marked as 
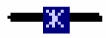
 and 
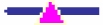
, respectively (n=8).

**Fig. 5:**
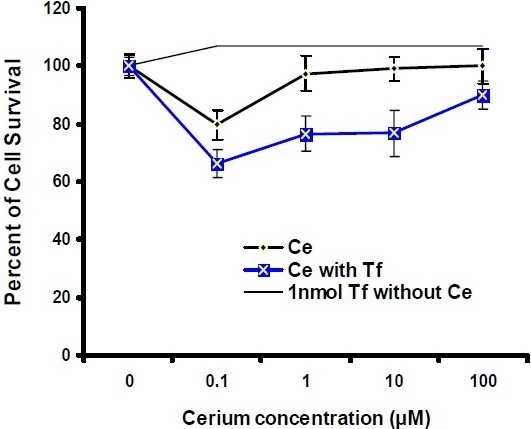
Percentage of MCF-7 cell survival after treatment with different concentrations of cerium (0, 0.1, 1, 10 and 100 μM), incubated for 48 h in the presence or absence of apo-transferrin (1 nM). The experiment in the absence and presence of transferrin is marked as 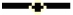 and 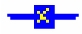, respectively (n=8). The upper solid line shows that the proliferation of MCF-7 cells increased with the presence of apo-transferrin (1 nM).

**Fig. 6:**
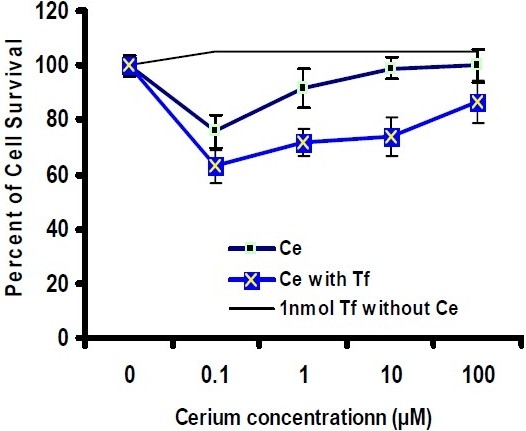
Percentage of MCF-7 cell survival after treatment with different concentrations of cerium (0, 0.1, 1, 10 and 100 μM), incubated for 48 h in the presence or absence of apo-transferrin (1 nM). The experiment in the absence and presence of transferrin is marked as 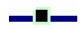 and 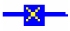, respectively (n=8). The upper solid line shows that the proliferation of MCF-7 cells increased with the presence of apo-transferrin (1 nM).

*In vitro* cytotoxicity assay was carried out in culture plates. The cells were allowed to settle by incubating the plates for 24 h before adding the compounds, and then were incubated for 48 h and 72 h with continuous exposure to cerium in the absence and presence of apo-transferrin at 37°C. The viability of Hela and MCF-7 cancer cells was then analyzed within the plates and compared with the control. As shown in Fig. 3 and 4, by increasing cerium concentrations, the percentage of cell survival of Hela cancer cells follows a descending trend line after 48 h and 72 h incubation period. Moreover, at 10 μM concentration of cerium, the inhibition activity was significantly induced by apo-transferrin (*P*<0.05). In similar conditions, these experiments were performed for MCF-7 breast cancer cells. The results showed that the trend lines for MCF-7 cells were completely different as compared with Hela cancer cells. MCF-7 cells were also treated with a serial concentration of cerium for 48 h and 72 h incubation periods. As shown in Fig. 5 and 6, at 1 μM and 10 μM concentration of cerium, the inhibition activity was significantly induced by apo-transferrin (*P*<0.05). The proliferation of MCF-7 cells was stimulated by apo-transferein alone, while this phenomenon was not observed for Hela cells. The significance of each cerium concentration with and without apo-transferrin in Hela and MCF-7 cell lines was compared to the control groups and examined by SPSS statistical software. The significance level was set at *P*<0.05.

## DISCUSSION

To determine the effect of cerium with and without transferrin on cell proliferation, a rapid colorimetric MTT assay was employed. This assay gives an objective for measurement of cell growth, since the formazan product is only formed in viable respiring cells. Practically, the linearity of the growth rate for Hela and MCF-7 cells were observed using a linear regression analysis. To understand *in vitro* growth characteristic for both cell lines, growth curves were plotted by formazan absorbance of viable cells against time (0, 24, 48, 72 and 96 h (Fig. 1 and 2). These results of in vitro chemosensitivity assessment of the cytotoxic agents indicated that the cells were actively proliferating during the period of compound administration.

Different expression levels of transferrin receptor in cancer cells, such as human epithelial cervical cancer cells and breast cancer cells, may promote cell proliferation and its malignancy(21,22). Transferrin is essential for transporting iron, which is required by all living organisms, especially highly proliferative cancer cells for DNA replication(22). Therefore, the primary role of transferrin is to promote cell growth by acting as an iron-dependent growth factor. As shown in Fig. 5 and 6, this phenomenon was observed for MCF-7 cells when the cells were treated only with apo-transferrin, while Hela cells could not respond significantly. Our results are consistent with the previous studies that transferrin enhanced cell proliferation in MCF-7 cancer cells(23,24). Cancer cells are characterized by cellular sources; as a result they display special activities in a defined pathway. Therefore, human epithelial cervical cancer cells could play a different manner compared with breast cancer cells.

In Hela cancer cells, at 10 μM concentration of cerium cell, the viability was reduced after 48 h and 72 h treatments, and in the presence of transferrin, the inhibitory effect of cerium on Hela cancer cell growth increased significantly (Fig. 3 and 4). Previously, a similar behavior was observed for MCF-7 cancer cell(25,26). When the cells were treated with serial concentrations of cerium without transferrin the cell growth was decreased. More experiments were performed with serial concentrations of cerium in the presence of transferrin. The cell viability significantly reduced especially at the concentration of 0.1 μM. Transferrin alone enhanced cell proliferation in MCF-7 cells; however, this phenomenon could not overcome the cerium inhibition activity on cell-proliferation. The biochemistry of cerium is similar to the biochemistry of ferric ion with regard to transferrin chelating, suggesting that this element probably enters the target cells with a mechanism similar to transferrin-receptor mediated endocytosis. The most important point that could be considered is that the cancer cells generally express very high transferrin receptors compared to the normal cells(27–29). Therefore, the malignant cell types are extremely dependent to iron ion for proliferation. In the presence of cerium and cerium-transferrin complex, the cell proliferation decreased significantly.

## CONCLUSION

Little is known about the effects of cerium in controlling cancer-cell growth. The biochemical similarities of cerium and iron ions, particularly regarding protein and ligand binding, help to predict the physiological role of cerium. In conclusion, although we could lay out the partial picture to describe how cerium ion and cerium-transferrin complex have inhibitory activity on cancer cell proliferation, the real mechanism of action remains unclarified. Further studies in these areas necessitate understanding the application of lanthanides in cancer therapy.
